# Reversal of Bortezomib-Induced Neurotoxicity by Suvecaltamide, a Selective T-Type Ca-Channel Modulator, in Preclinical Models

**DOI:** 10.3390/cancers13195013

**Published:** 2021-10-07

**Authors:** Cristina Meregalli, Yuri Maricich, Guido Cavaletti, Annalisa Canta, Valentina A. Carozzi, Alessia Chiorazzi, Evan Newbold, Paola Marmiroli, Cecilia Ceresa, Arthur Diani, Spyros Papapetropoulos, Margaret S. Lee

**Affiliations:** 1 Experimental Neurology Unit, School of Medicine and Surgery and NeuroMI (Milan Center for Neuroscience), University of Milano Bicocca, 20900 Monza, Italy; cristina.meregalli@unimib.it (C.M.); guido.cavaletti@unimib.it (G.C.); annalisa.canta@unimib.it (A.C.); valentina.carozzi1@unimib.it (V.A.C.); alessia.chiorazzi@unimib.it (A.C.); paola.marmiroli@unimib.it (P.M.); cecilia.ceresa@gmail.com (C.C.); 2 Pear Therapeutics, 200 State Street, Boston, MA 02019, USA; yuri@maricich.org; 3 Jazz Pharmaceuticals, 2005 Market Street, Suite 2100, Philadelphia, PA 19103, USA; enewbold54@gmail.com; 4 Department of Biotechnology and Bioscience, University of Milano Bicocca, 20900 Monza, Italy; 5 212 Wild Oak Drive, Swansboro, NC 28584, USA; arthur.r.diani@gmail.com; 6 Massachusetts General Hospital, 55 Fruit Street, Boston, MA 02114, USA; spapapetropoulos@gmail.com

**Keywords:** suvecaltamide, CX-8998, JZP385, non-interference, reversal, bortezomib, neurotoxicity, chemotherapy-induced peripheral neurotoxicity

## Abstract

**Simple Summary:**

Chemotherapy-induced peripheral neurotoxicity (CIPN) is a side-effect of anti-cancer medications, which can lead to pain, disruptions to movement, and eventually results in the need to interrupt or stop chemotherapy. This study sought to test whether the drug suvecaltamide could help to reduce the impact of the chemotherapy agent bortezomib (BTZ) on symptoms of CIPN using animal models and human cells. Suvecaltamide did reverse negative changes in nerve conduction velocity and intraepidermal nerve fiber density indicative of CIPN in rats, and did not interfere with the anti-cancer effect of BTZ. These results indicate that suvecaltamide could potentially be useful for patients experiencing CIPN, although further mechanistic and molecular studies in vitro and in vivo are required before clinical trials.

**Abstract:**

This study evaluated suvecaltamide, a selective T-type calcium channel modulator, on chemotherapy-induced peripheral neurotoxicity (CIPN) and anti-cancer activity associated with bortezomib (BTZ). Rats received BTZ (0.2 mg/kg thrice weekly) for 4 weeks, then BTZ alone (*n* = 8) or BTZ+suvecaltamide (3, 10, or 30 mg/kg once daily; each *n* = 12) for 4 weeks. Nerve conduction velocity (NCV), mechanical threshold, β-tubulin polymerization, and intraepidermal nerve fiber (IENF) density were assessed. Proteasome inhibition was evaluated in peripheral blood mononuclear cells. Cytotoxicity was assessed in human multiple myeloma cell lines (MCLs) exposed to BTZ alone (IC_50_ concentration), BTZ+suvecaltamide (10, 30, 100, 300, or 1000 nM), suvecaltamide alone, or vehicle. Tumor volume was estimated in athymic nude mice bearing MCL xenografts receiving vehicle, BTZ alone (1 mg/kg twice weekly), or BTZ+suvecaltamide (30 mg/kg once daily) for 28 days, or no treatment (each *n* = 8). After 4 weeks, suvecaltamide 10 or 30 mg/kg reversed BTZ-induced reduction in NCV, and suvecaltamide 30 mg/kg reversed BTZ-induced reduction in IENF density. Proteasome inhibition and cytotoxicity were similar between BTZ alone and BTZ+suvecaltamide. BTZ alone and BTZ+suvecaltamide reduced tumor volume versus the control (day 18), and BTZ+suvecaltamide reduced tumor volume versus BTZ alone (day 28). Suvecaltamide reversed CIPN without affecting BTZ anti-cancer activity in preclinical models.

## 1. Introduction

Bortezomib (BTZ), a chemotherapeutic agent that acts as a proteasome inhibitor in multiple myeloma cells, is used clinically in patients with newly diagnosed and relapsed multiple myeloma [1]. In clinical trials, BTZ provided benefits including greater overall response rates, slower progression, and increased overall survival rates [2,3,4,5].

Chemotherapy-induced peripheral neurotoxicity (CIPN) is a side effect of anti-cancer agents, including BTZ [1,6,7,8,9,10]. CIPN negatively impacts motor and sensory neurons and causes nerve fiber degeneration [11,12,13,14]. Symptoms include pain, numbness, burning of extremities, and motor dysfunction, potentially leading to chemotherapy dose reduction/termination and shortened survival [15].

T-type calcium channels (TTCCs) Cav3.1, Cav3.2, and Cav3.3 regulate neuronal excitability [16,17,18,19] and have emerged as targets for treating CIPN. TTCCs are highly expressed in primary sensory neurons [20]. Overactivation or increased expression of TTCCs results in chronic pain [20,21,22], while the blockade of TTCCs reduces painful behavior in animal models [23,24,25]. BTZ elevates Cav3.2 protein levels and calcium currents in TTCCs of afferent neurons by inhibiting proteasome degradation, a pathophysiologic mechanism linked to CIPN [26].

Suvecaltamide (also known as JZP385, and formerly known as CX-8998) is a potent and selective modulator of TTCCs that exhibits a much higher affinity for the inactivated channel conformation, suggesting that it state-dependently blocks calcium channels under conditions of electrical excitability [27]. In preclinical and clinical studies conducted to date, suvecaltamide was well tolerated, demonstrating a favorable safety profile [28,29].

The goals of this study were to evaluate the effects of suvecaltamide on BTZ-induced CIPN and to determine whether suvecaltamide interferes with BTZ anti-cancer activity (proteasome inhibition, cytotoxicity, and anti-tumor activity) in rodent models and human multiple myeloma cell lines (MCLs).

## 2. Methods

### 2.1. In Vivo BTZ-Induced CIPN Reversal Study

#### 2.1.1. BTZ-Induced CIPN Rat Model

Care and husbandry for the Wistar rat study conformed to the University of Milano Bicocca guidelines and complied with national (D.L.vo n. 26/2014) and international regulations and policies (Directive 2010/63/EU). The protocol (47123/14) was approved by the University of Milano Bicocca Ethics Committee. Rats were housed in Makrolon cages (220 × 390 × 180 mm; Tecniplast Gazzada, Varese, Italy) and maintained on 12 mm Global Diet Pellets 2018 (Mucedola srl, Settimo Milanese, Italy).

Female Wistar rats (*n* = 52) (Envigo, Udine, Italy) at 10–11 weeks of age were utilized. Rats were housed 2–3 per cage under controlled environmental conditions (22 ± 2 °C, 55 ± 10% relative humidity) and 12-h light/dark cycles (07:00 a.m.–19:00 p.m.). The study was divided into two phases of 4 weeks each. In phase 1, rats were randomized into two groups, one that was untreated (CTRL, *n* = 8) and one that received BTZ 0.2 mg/kg (vehicle: 10% Tween^®^ 80, 10% ethanol 100, and saline) intravenously (IV) via the tail vein, thrice weekly, for 4 weeks (BTZ, *n* = 44). The dose of BTZ was based on previous experiments [30]; moreover, the control animals were untreated, as the toxicity of the BTZ vehicle was not evident in previously published data [31]. Body weight and animal observations were evaluated twice weekly during phase 1 from baseline (day 1) to day 28. At baseline and at the end of phase 1 (day 28), nerve conduction velocity (NCV; caudal and sciatic nerves) and mechanical threshold (MT; hind paw) were measured. Blood samples were collected 1 h after the administration of BTZ on day 1 for the proteasome inhibition assays.

In phase 2, BTZ-treated rats were divided into four groups: one group (*n* = 8) received BTZ 0.2 mg/kg IV thrice weekly for 4 weeks, and the remaining 3 groups (*n* = 12 each) received co-treatment with BTZ 0.2 mg/kg thrice weekly and suvecaltamide 3, 10, or 30 mg/kg (vehicle: 1% Tween 80, 99% Methocel 0.5%) by oral gavage daily for 4 weeks (BTZ+suvecaltamide 3, BTZ+suvecaltamide 10, and BTZ+suvecaltamide 30, respectively). The untreated CTRL rats from phase 1 remained untreated in phase 2. The suvecaltamide doses selected for this study were utilized in prior preclinical studies and have been shown to be well tolerated. In addition, these doses have been shown to result in clinically relevant plasma concentrations. In phase 2, body weight was measured twice weekly from baseline (day 28) to day 56 (end of treatment). NCV and MT were measured at baseline and on days 35 and 56. Blood samples were collected 1 h after the administration of BTZ on days 28, 35, and 56 for proteasome inhibition assays. At the end of phase 2, sciatic nerves were obtained for β-tubulin polymerization, as well as skin samples for evaluation of the intraepidermal nerve fiber (IENF) density and histopathology. Differences in body weight, NCV, MT, β-tubulin polymerization, IENF density, and proteasome inhibition were analyzed by the Mann−Whitney test for comparison between the control and BTZ groups at the end of the 4-week treatment period, and then with the Kruskal−Wallis and Dunn’s multiple comparison post-test for comparison between all groups at the 5- and 8- week time points.

#### 2.1.2. NCV Measurement

NCV (meters/second) was obtained from caudal and sciatic nerves using an electromyography tool (Myto 2, ABN Neuro, Firenze, Italy). Caudal NCV was measured through placement of the recording needle electrodes distally in the tail with stimulating needle electrodes 5 cm and 10 cm proximal to the recording point. Peak latencies of potentials recorded at the two sites after nerve stimulation were determined and NCV was calculated. Sciatic NCV was determined by placement of needle recording electrodes near the ankle bone and stimulating electrodes close to the thigh. As with the caudal nerve, peak latencies were recorded and NCV was calculated. NCV was performed under standard conditions in a temperature-controlled facility (22 ± 2 °C) while rats were under isoflurane anesthesia with monitoring of the vital signs. The positions of the stimulating and recording electrodes were the same across each of the recording days for each rat.

#### 2.1.3. MT Measurement

MT was assessed with the Dynamic Aesthesiometer Test device (Model 37450; Ugo Basile Biological Instruments, Comerio, Italy). After acclimation, a servo-controlled pointed metallic filament (0.5 mm diameter) placed on the plantar surface of the hind paw exerted a progressive punctate pressure up to 50 g within 20 s. The pressure elicited a voluntary hind paw withdrawal response that was recorded and represented the MT index. MT was collected alternatively on each hind paw every 2 min on three occasions to yield a mean value. Mean MT values represented maximum pressure (grams) tolerated by each rat. Exposure of each animal to the mechanical stimulus was limited to 20 s.

#### 2.1.4. Tissue Sample Collection

At day 56, rats were sacrificed by CO_2_ inhalation, cervical dislocation was performed, and tissue samples were procured from four randomly selected rats per group. Right sciatic nerves were frozen in liquid nitrogen for β-tubulin polymerization assay, and plantar glabrous skin samples were collected for IENF density.

#### 2.1.5. β-Tubulin Polymerization Assay

Protein extracts from sciatic nerves were solubilized in a lysis buffer (10% glycerol; 25 mM TRIS-HCl, pH 7.5; 1% Triton X-100; 5 mM EDTA, pH 8; and 1 mM EGTA, pH 8) containing freshly added protease and phosphate inhibitors (10 mM sodium orthovanadate, 4 mM phenylmethylsulfonyl fluoride, 1% aprotinin, and 20 mM sodium pyrophosphate). Protein extracts were centrifuged (14,000 rpm for 10 min at 4 °C) to separate the soluble (S) free tubulin fractions from polymerized (P) fractions. Supernatants (S) were collected and pellets (P) of polymerized tubulin were resuspended by sonication for 20 s in a lysis buffer supplemented with 0.5% sodium deoxycholate. Protein aliquots (10 µg) were placed onto 13% SDS-PAGE and, after electrophoresis, were transferred to nitrocellulose filters. Immunoblotting analysis was performed using a mouse anti-β-tubulin antibody (Sigma, Milano, Italy). After incubation with the primary antibody, the membrane was washed and incubated with horseradish peroxidase conjugated to goat anti-rabbit IgG (Perkin Elmer Italia SPA, Monza, Italy). The ECL chemiluminescence system (Amersham GE Healthcare Europe GmbH, Milano, Italy) was used for detection. Band intensity was quantified with Gel Logic 100 Image System (Eastman Kodak, Rochester, NY, USA). The final mean values were obtained from triplicate experiments and data were expressed as percentage of P/P+S in treated rats compared with the controls.

#### 2.1.6. IENF Density

Plantar glabrous skin samples (5 mm) from rat hind paws were fixed in 2% PLP (paraformaldehyde−lysine−sodium periodate) solution for 24 h at 4 °C and were cryoprotected overnight. Samples were serially cut with a cryostat to yield 20 µm sections. Three sections from each footpad were randomly selected and immunostained with rabbit polyclonal anti-protein gene product 9.5 (PGP 9.5; Bio-Rad AbD Serotec Ltd., Oxford, UK) in combination with biotinylated anti-rabbit IgG and Vector SG substrate kit peroxidase (Vector Laboratories, Burlingame, CA, USA) using a free-floating protocol. The same blinded observer (ACh) counted the total number of immunopositive IENF in each section under light microscopy at high magnification with a microscope video camera. Individual fibers that crossed the dermal−epidermal interface were counted. Secondary branches within epidermis were excluded. The length of the epidermis was measured to generate the linear density of IENF/millimeter [32].

#### 2.1.7. Proteasome Inhibition Assay

Peripheral blood mononuclear cells (PBMCs) were isolated from blood samples by Ficoll-Hypaque density separation. Cells were added to the lysis solution (50 mM HEPES, 5 mM EDTA, 150 mM NaCl and Triton-X100 1% in water) and extracted. Lysates were centrifuged at 13,500 rpm for 15 min at 4 °C. Protein extracts were processed similar to the β-tubulin polymerization assay, except that the protease and phosphate inhibitors were not added to the lysis buffer, and were centrifuged at 14,000 rpm for 10 min at 4 °C. Protein concentration was assessed by the Bradford assay with a Coomassie^®^ Protein Assay Reagent Kit (Pierce, Thermo Scientific, Rockford, IL, USA). A fluorometric assay evaluated the proteasomal activity and the protein extract was incubated with N-succinyl-Leu-Leu-Val-Tyr-7-Amido-4-Methylcoumarin substrate (Sigma Aldrich, Milano, Italy) for 2 h. Proteasome activity was detected as a relative light unit generated from cleaved substrate in the reagent. Fluorescence (F) from each reaction was assessed with a fluorometer (Wallac 1420 multilabel counter, Perkin Elmer Italia SPA, Monza, Italy). The proteasome activity (PA) was calculated as % PA = (F BTZ-F Substrate)/(F Control-F Substrate) and inhibition was expressed as 100 × (1-PA).

### 2.2. In Vitro BTZ Cytotoxicity Study

#### 2.2.1. Chemicals and Drugs

Roswell Park Memorial Institute (RPMI) 1640 medium, penicillin (100 U/mL), streptomycin (100 µg/mL), HEPES (4-(2-hydroxyethyl)-1-piperazineethanesulfonic acid), sodium bicarbonate, and sodium pyruvate were purchased from EuroClone SpA (Pero, Italy). Fetal bovine serum (FBS) was procured from Hyclone Laboratories, Inc. (Logan, UT, USA). All other chemicals were obtained from Sigma-Aldrich (St. Louis, MO, USA). BTZ was acquired from LC Laboratories (Woburn, MA, USA) and suvecaltamide was provided by Cavion, Inc. (Charlottesville, VA, USA). BTZ (2.6 mM) and suvecaltamide (10 mM) were dissolved in dimethyl sulfoxide (DMSO) and diluted in the culture medium.

#### 2.2.2. MCLs

RPMI 8226 cells were gifted from Dr. Catia Morelli (University of Calabria, Catanzaro, Italy). MM.1S and U266B1 cells were obtained from the American Type Culture Collection (LGC Standards, Sesto San Giovanni, Italy). All cell lines were maintained in floating culture with an RPMI medium containing 2 mM L-glutamine supplemented with 10% FBS, penicillin, and streptomycin. MM.1S cell medium was supplemented with 1.5 g/L sodium bicarbonate, 10 mM HEPES, and 1 mM sodium pyruvate. The cells were grown in 75 cm^2^ culture flasks for floating cells (Corning Inc. Corning, NY, USA) at 37 °C in 5% CO_2_ and 95% air.

#### 2.2.3. Cell Survival Assay

The sulforhodamine B assay was used to measure the cell growth inhibitory effect (percent cell survival) of BTZ. Three human MCLs (RPMI 8226, MM.1S, and U266B1) were plated in 96-well plates (Eppendorf, Milano, Italy) at 10,000 cells/well. After 24 h, cells were exposed to BTZ (0.05–250 nM) for 72 h. Eight BTZ doses (0.05, 0.1, 0.5, 5, 10, 50, 100, and 250 nM) were used to obtain a dose−response curve and calculate the 50% inhibitory concentration (IC_50_). BTZ was diluted in a culture medium for testing. BTZ was removed after the 72-h treatment, and then the SRB assay was performed. Cells were fixed with trichloroacetic acid for 1 h and stained for 15 min with sulforhodamine B dissolved in 1% acetic acid. Unbound dye was removed by five washes with 1% acetic acid. Bound dye was dissolved with a solution of Tris (hydroxymethyl) aminomethane base and absorbance was measured at 540 nM. Growth inhibition was expressed as a percentage of DMSO control absorbance of the cells, corrected for absorbance before the addition of the drug. The IC_50_ of percent cell survival by BTZ versus the control was calculated by nonlinear least squares curve fitting with Prism software (version 4.0, GraphPad Software Inc., La Jolla, CA, USA).

To assess the effect of suvecaltamide on the cell growth inhibitory effect of BTZ, the MCLs were exposed for 72 h to the IC_50_ concentration of BTZ alone or in combination with each of five different concentrations (10, 30, 100, 300, or 1000 nM) of suvecaltamide. These concentrations (over two orders of magnitude) of suvecaltamide were based on previous unpublished cell culture experiments. Incubations with each of the five concentrations of suvecaltamide alone and the DMSO control alone were also performed. Growth inhibition of each of the three cell lines was measured by the sulforhodamine B assay and expressed as mean ± SD percent cell survival for each concentration of BTZ or BTZ+suvecaltamide versus control (control value of 100%). Differences in percent cell survival between the BTZ, BTZ+suvecaltamide, and control were analyzed using non-linear least squares analyses.

#### 2.2.4. In Vivo BTZ Anti-Tumor Activity Study

South Texas Accelerated Research Therapeutics (START) (San Antonio, TX, USA) conducted this study. Care and husbandry of the mice complied with the United States Department of Agriculture (USDA) Animal Welfare Act and the START Institutional Animal Care and Use Committee (IACUC) regulations. The protocol was reviewed and approved by the START IACUC. Mice were individually housed in Sealsafe^®^ Plus ventilated cages (Tecniplast, West Chester, PA, USA) and fed with Teklad 2919 (Envigo, Somerset, NJ, USA), irradiated, 19% protein, 9% fat, and 4% fiber mouse chow. Mice were maintained under controlled environmental conditions similar to those used in the rat study (22 ± 2 °C, 55 ± 10% relative humidity, and 12-h light/dark cycles (07:00 a.m.–19:00 p.m.)).

BTZ alone and the combination of BTZ+suvecaltamide were tested for anti-tumor activity in a START cell-based xenograft (START-CBX) athymic nude mouse (Crl: NU(NCr)-Foxn1^nu^) tumor model of human myeloma. RPMI-8226 cells were subcutaneously injected (10^5^ cells) into 32 female athymic nude mice (Charles River Laboratories, Houston, TX, USA) at 6–12 weeks of age. The study was initiated when the tumor volume reached 125–250 mm^3^. Tumor volume was estimated by measurement of palpable mass with a digital caliper and expressed in mm^3^ with the formula width^2^ × length × 0.52. Mice were stratified by mean tumor volume into four groups of eight animals each: tumor vehicle control (0.5% methylcellulose, 1% Tween-80 orally once daily for 18 days), non-tumor control (no treatments for 28 days; i.e., no IV or oral vehicle), BTZ alone (BTZ 1 mg/kg IV twice weekly for 28 days; no oral vehicle), and BTZ+suvecaltamide (BTZ 1 mg/kg IV and suvecaltamide 30 mg/kg orally once daily for 28 days). Body weight, tumor volume, and animal observations were collected twice weekly up until day 18 (termination) for the tumor vehicle control mice, and twice weekly to day 28 (termination) for the other three groups. Mean ± SD body weight and tumor volume were analyzed with the Kruskal−Wallis and Dunn multiple comparison test at day 18, and with the Mann−Whitney test at day 28.

### 2.3. Ethical Standards

As detailed above, all study protocols were approved by the relevant institutional ethics review boards. The care and handling of animals were conducted in compliance with institutional and national standards.

### 2.4. Statistical Analysis

Statistical methods are detailed above. *p* values are nominal and are reported in Table S1.

## 3. Results

### 3.1. Effect of Suvecaltamide on BTZ-Induced CIPN

The effect of suvecaltamide on BTZ-induced CIPN was evaluated in a rat model using a two-phase study design. At the end of phase 1 (week 4), there was no difference in body weight (grams) between the BTZ and CTRL groups. After re-randomization and an additional 1 or 4 weeks of treatment (weeks 5 and 8, respectively), body weight changes were comparable in all of the analyzed groups. Treatment with BTZ alone and in combination with suvecaltamide was tolerated based on body weight change and behavior, even though two animals (in the BTZ+suvecaltamide 3 and BTZ+suvecaltamide 10 groups) died before the end of the experiment.

#### 3.1.1. NCV and MT

Reduced NCV and mechanical allodynia (which can be measured via MT) are characteristic of BTZ-induced neurotoxicity in rats [12,30].

**Figure 1:**
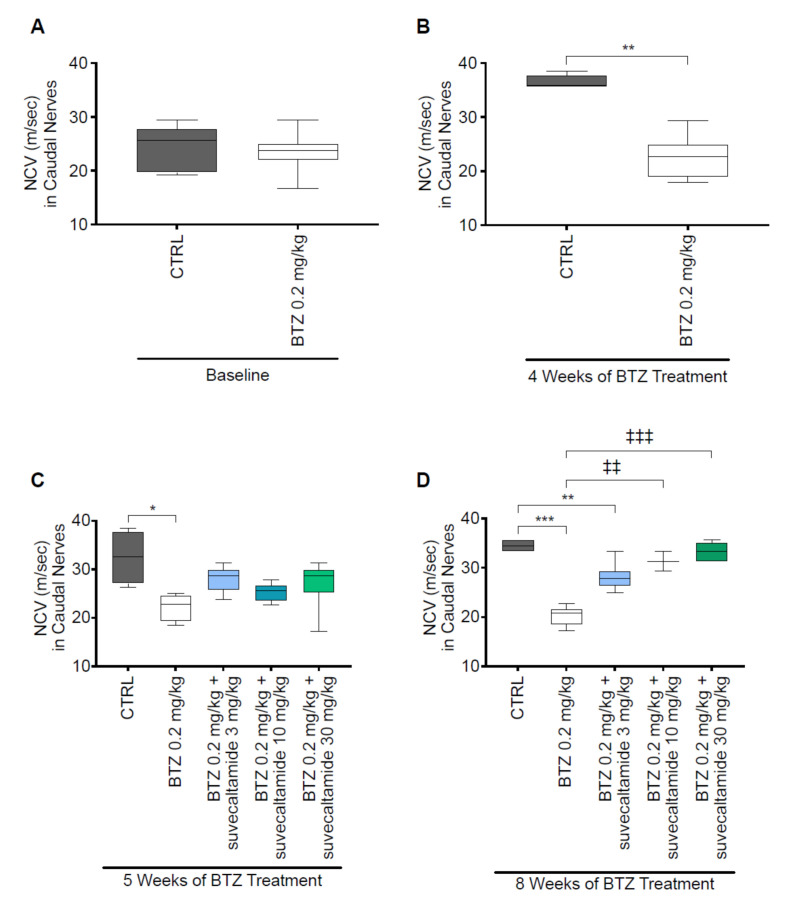
Caudal nerve conduction velocity (NCV). Conduction velocity obtained from caudal nerves by electromyography during phase 1 ((**A**) baseline and (**B**) 4 weeks) and phase 2 ((**C**) 5 and (**D**) 8 weeks) in a rat model of BTZ-induced CIPN. BTZ—bortezomib; CIPN—chemotherapy-induced peripheral neurotoxicity; CTRL—untreated animals. * *p* < 0.05 vs. CTRL; ** *p* < 0.01 vs. CTRL; *** *p* < 0.001 vs. CTRL; ^‡‡^ *p* < 0.01 vs. BTZ; ^‡‡‡^ *p* < 0.001 vs. BTZ.

**Figure 2:**
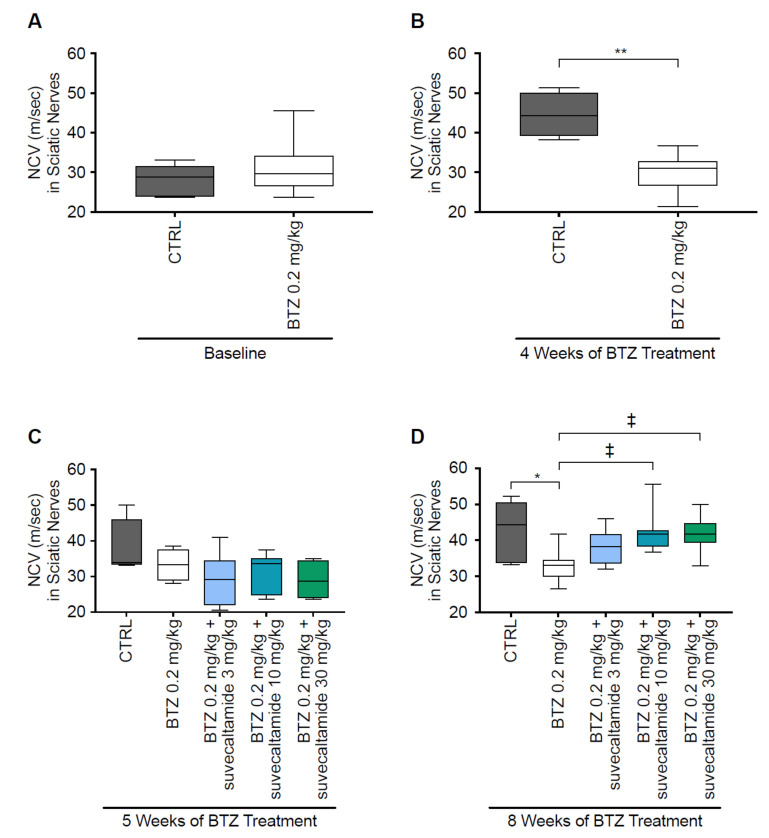
Sciatic nerve conduction velocity (NCV). Conduction velocity obtained from sciatic nerves by electromyography during phase 1 ((**A**) baseline and (**B**) 4 weeks) and phase 2 ((**C**) 5 and (**D**) 8 weeks) in a rat model of BTZ-induced CIPN. BTZ—bortezomib; CIPN—chemotherapy-induced peripheral neurotoxicity; CTRL—untreated animals. * *p* < 0.05 vs. CTRL; ** *p* < 0.01 vs. CTRL; ^‡^ *p* < 0.05 vs. BTZ.

At baseline (day 1), NCV (meters/second) of caudal (Figure 1A; Table S1) and sciatic (Figure 2A and Table S1) nerves were similar between the CTRL and BTZ alone groups. NCV was reduced by BTZ alone versus CTRL at weeks 4 and 5 for caudal nerves (*p* < 0.01 and *p* < 0.05, respectively; Figure 1B,C) and at week 4 for sciatic nerves (*p* < 0.01; Figure 2B,C). At week 5, there was no statistically significant difference in the NCV of sciatic nerves between BTZ alone and CTRL (*p* > 0.05). The greater overall variability in sciatic nerve NCV values, smaller number of animals per group, and/or lower absolute effect of BTZ alone on reducing NCV at week 5 versus week 8 may have contributed to an inability to detect differences. At week 8, suvecaltamide dose-dependently increased NCV, with statistically significant differences noted between the highest doses of suvecaltamide (10 or 30 mg/kg) in combination with BTZ versus BTZ alone in caudal nerves (*p* < 0.01 and *p* < 0.001, respectively; Figure 1D) and sciatic nerves (*p* < 0.05 for both comparisons; Figure 2D). At the highest dose of suvecaltamide (30 mg/kg), NCV levels in caudal and sciatic nerves were similar to the levels in the CTRL group.

**Figure 3:**
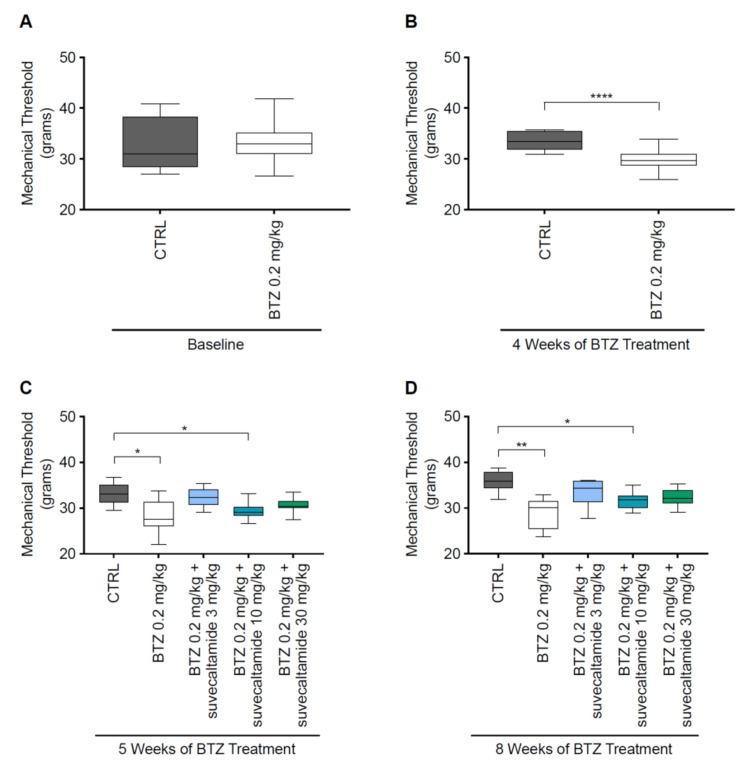
Mechanical threshold (MT). Evaluation of mechanical allodynia measured using a Dynamic Aesthesiometer Test during phase 1 ((**A**) baseline and (**B**) 4 weeks) and phase 2 ((**C**) 5 and (**D**) 8 weeks) in a rat model of BTZ-induced CIPN. BTZ—bortezomib; CIPN—chemotherapy-induced peripheral neurotoxicity; CTRL—untreated animals. * *p* < 0.05 vs. CTRL; ** *p* < 0.001 vs. CTRL; **** *p* = 0.0001 vs. CTRL.

At baseline (day 1), MT (grams) in the hind paws was comparable between the CTRL and BTZ alone groups (Figure 3A). At weeks 4, 5, and 8, MT was reduced (*p* = 0.0001, *p* < 0.05, and *p* < 0.001, respectively; Figure 3B–D) with BTZ alone versus CTRL, indicating the development of mechanical allodynia. There were no significant differences between BTZ alone versus BTZ+suvecaltamide (any concentration) at weeks 5 and 8 (*p* > 0.05 for all comparisons; Table S1).

#### 3.1.2. Tissue Assessments

Elevated β-tubulin polymerization [33,34] and decreased IENF density are tissue abnormalities associated with BTZ-induced neurotoxicity [12,30].

**Figure 4:**
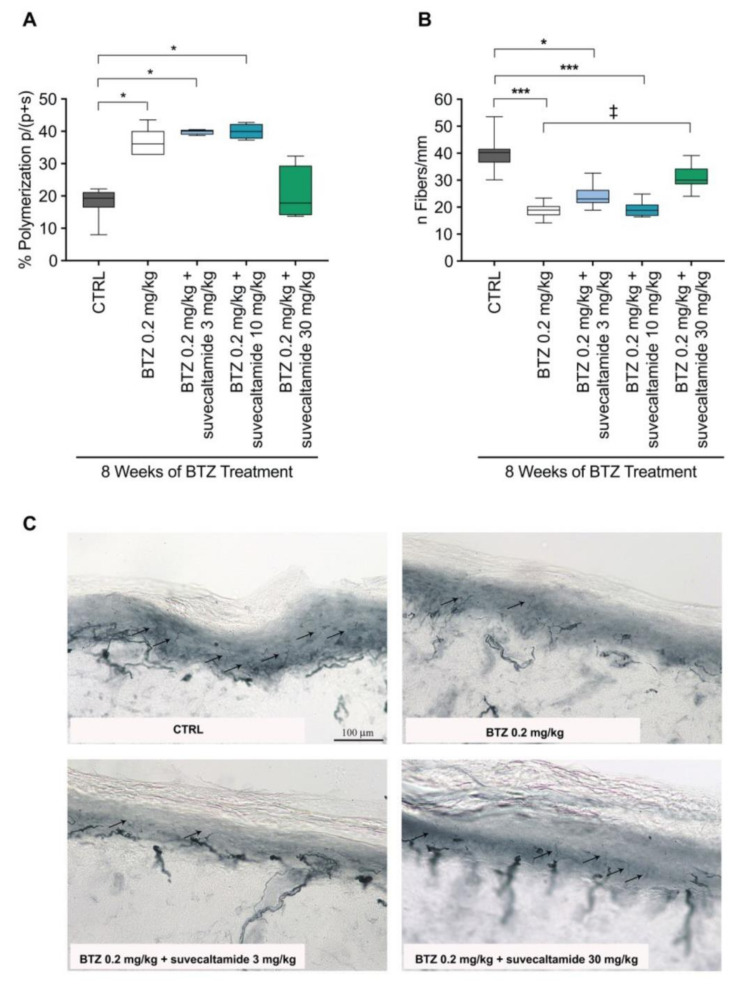
*β*-Tubulin polymerization, intraepidermal nerve fiber density (IENF), and histopathology. (**A**) Percent of β-tubulin polymerization in protein extracts from sciatic nerve tissue collected after 8 weeks of treatment with BTZ in the presence or absence of suvecaltamide. (**B**) Number of nerve fibers per mm quantified in sections of plantar glabrous skin from hind paws collected after 8 weeks of treatment with BTZ in the presence or absence of suvecaltamide. (**C**) Representative images of tissue samples quantified in panel B. BTZ—bortezomib; CTRL—untreated animals. * *p* < 0.05 vs. CTRL; *** *p* < 0.001 vs. CTRL; ^‡^ *P* < 0.05 vs. BTZ.

β-Tubulin polymerization (%) in sciatic nerves was increased with BTZ alone and BTZ+suvecaltamide (3 and 10 mg/kg) versus CTRL at week 8 (both *p* < 0.05; Figure 4A). There was a trend toward the reversal of this increase with BTZ+suvecaltamide 30 mg/kg (*p* > 0.05 versus BTZ alone and CTRL; Figure 4A, Table S1).

IENF density (number of fibers/mm) in hind paw tissue samples was decreased with BTZ alone versus CTRL at week 8 (*p* < 0.001; Figure 4B). This decrease was reversed with BTZ+suvecaltamide 30 mg/kg (*p* < 0.05 versus BTZ alone; Figure 4B, Table S1).

Qualitative light microscopic analysis of the nerve fibers in the plantar glabrous skin of the hind paw suggested that more nerve fibers were evident with BTZ+suvecaltamide 30 mg/kg versus BTZ alone (Figure 4C), and these qualitative nerve fiber observations are consistent with IENF density data (Figure 4B).

### 3.2. Effect of Suvecaltamide on BTZ Anti-Cancer Activity

#### 3.2.1. Proteasome Inhibition

**Figure 5:**
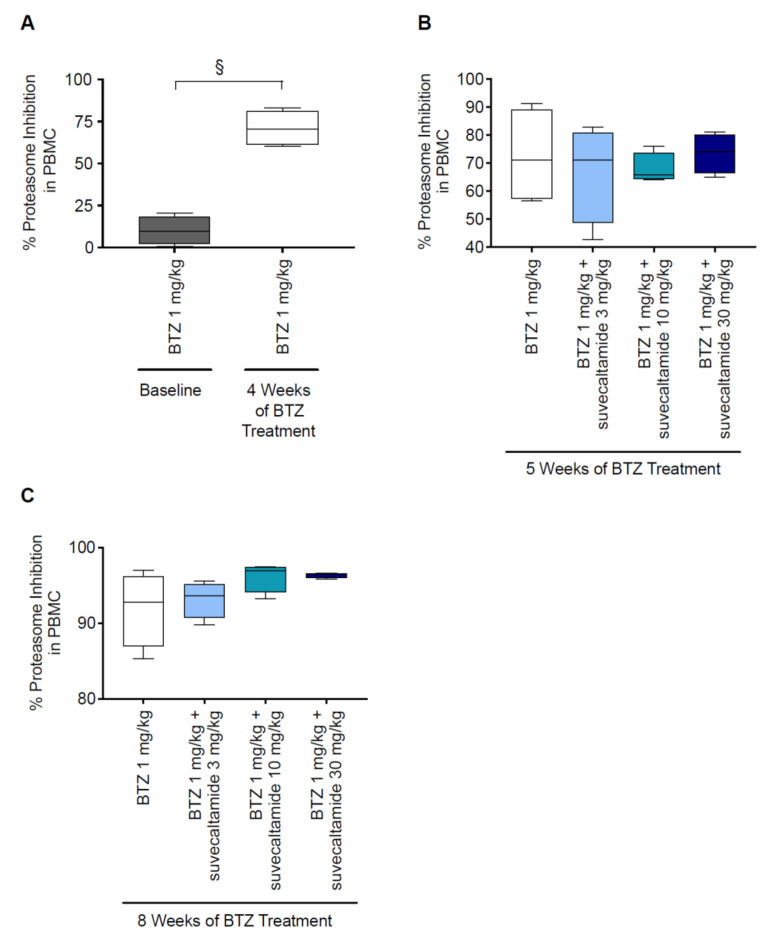
Proteasome inhibition. Percent proteasome inhibition in PBMCs isolated from rats in the BTZ-induced CIPN rat model at (**A**) 4 weeks, (**B**) 5 weeks, and (**C**) 8 weeks. BTZ—bortezomib; CIPN—chemotherapy-induced peripheral neurotoxicity; PBMC—peripheral blood mononuclear cell. ^§^ *p* < 0.05 vs. BTZ baseline.

BTZ treatment resulted in a greater inhibition of % proteasome activity in circulating PBMCs at week 4 versus baseline (*p* < 0.05; Figure 5A, Table S1). At weeks 5 and 8, the proteasome activity was similarly inhibited by BTZ alone and BTZ+suvecaltamide (all doses; Figure 5B,C). These data suggest that the co-administration of suvecaltamide did not interfere with the anti-proteasome activity of BTZ.

#### 3.2.2. Cytotoxicity

BTZ caused a concentration-dependent inhibition of cell growth (cytotoxicity) in the three MCLs. IC_50_ BTZ values were 6 ± 0.5 nM, 4 ± 1.7 nM, and 2.5 ± 0.6 nM for the MM.1S, RPMI 8226, and U266B1 cell lines, respectively.

**Figure 6:**
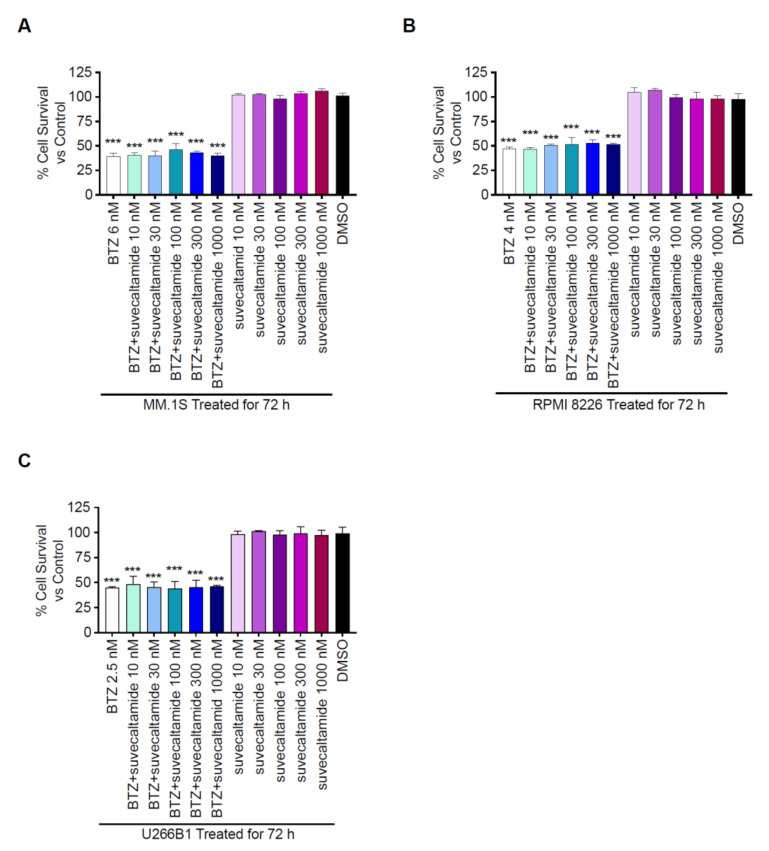
Cytotoxicity. Percent survival of MCLs: (**A**) MM.1S, (**B**) RPMI 8226, and (**C**) U266B1 treated in vitro for 72 h with BTZ at the IC50 (6 ± 0.5 nM, 4 ± 1.7 nM, and 2.5 ± 0.6 nM, respectively) in the presence or absence of various concentrations of suvecaltamide. BTZ—bortezomib; MCL—human multiple myeloma cell line. *** *p* < 0.001 vs. control.

BTZ alone (at IC_50_ concentrations) reduced percent cell survival of the three MCLs versus the DMSO control (*p* < 0.001; Figure 6). BTZ+suvecaltamide (10–1000 nM) also reduced the percent cell survival of the three MCLs (*p* < 0.001) versus the DMSO control; the magnitude of percent cell survival reduction after treatment with BTZ+suvecaltamide appeared similar to that with BTZ alone (Figure 6). Suvecaltamide alone did not reduce the percent cell survival in any of the MCLs versus the DMSO control (Figure 6).

#### 3.2.3. Anti-Tumor Activity

**Figure 7:**
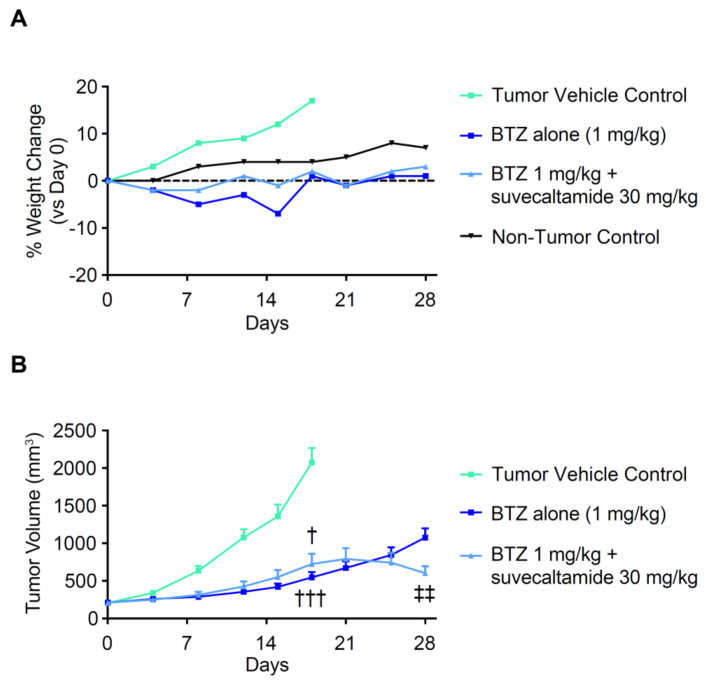
Body weight and tumor volume. (**A**) Relative body weight and (**B**) tumor volume in athymic nude mice bearing RPMI8226 xenografts. BTZ—bortezomib; tumor vehicle control (0.5% methylcellulose, 1% tween-80). ^†^ *p* < 0.05 vs. vehicle control; ^†††^ *p* < 0.001 vs. tumor vehicle control; ^‡‡^ *p* < 0.01 vs. BTZ.

The impact of suvecaltamide on BTZ anti-tumor activity in vivo was evaluated in athymic nude mice bearing RPMI-8229 human MCL xenografts. Mean body weight at baseline was 19.9 g, 20.9 g, 20.2 g, and 21.3 g in the tumor vehicle control, BTZ, BTZ+suvecaltamide, and non-tumor control groups, respectively. From baseline (day 0) to day 18, the percent body weight gain was more evident in the vehicle control group (consistent with the tumor xenograft growth) versus the non-tumor control group (normal animal growth; Figure 7A). Treatment with BTZ alone or BTZ+suvecaltamide 30 mg/kg resulted in transient body weight loss during the first 2 weeks of treatment and no weight gain at days 18 and 28, consistent with the known anti-tumor and weight loss effects of BTZ in this model (Figure 7A).

At day 18, the tumor volume was reduced with BTZ alone and BTZ+suvecaltamide versus the vehicle control (*p* < 0.001 and *p* < 0.05, respectively; Figure 7B, Table S1). The rate of tumor volume growth from day 18 through day 28 was greater in the mice treated with BTZ alone versus BTZ+suvecaltamide. In the BTZ+suvecaltamide group, tumor volume growth appeared to plateau and then decreased at day 28 compared with BTZ alone (between-group difference, *p* < 0.01 at termination; Figure 7B).

Overall, in vivo tumor volume and weight gain data and in vitro cell survival data were convergent, and suggest that suvecaltamide does not block or attenuate the anti-tumor activity or weight loss effect of BTZ.

## 4. Discussion

The studies reported here examined the effects of suvecaltamide, a potent and selective TTCC modulator, on BTZ-induced CIPN and BTZ anti-cancer activity in rodent models and human MCLs. BTZ is an FDA-approved, first-line chemotherapeutic agent indicated for patients with newly diagnosed and relapsed multiple myeloma, which acts as a selective, reversible boronic acid dipeptidase inhibitor of the 20S proteasome protein complex in multiple myeloma secretory cells [10]. These cells are prime targets for proteasome inhibition because of their increased protein processing [35]. Proteasome inhibition is a critical mechanism for anti-cancer activity because it leads to the disassembly of anti-apoptotic proteins and protects against the breakdown of pro-apoptotic proteins, both of which stimulate the death of malignant cells [2]. Pivotal multiple myeloma clinical trials have shown that BTZ alone or as part of combination therapy provided clinically significant benefits, including greater overall response rates, longer time to disease progression, and increased overall survival rates [2,3,4,5]. BTZ treatment is associated with peripheral neuropathy, potentially leading to dose adjustment to alleviate symptoms [1,10]. Currently, there are no approved pharmacotherapies for the prevention or reversal of BTZ-induced CIPN. The American Society of Clinical Oncology has recommended only a single drug, duloxetine, for treatment of CIPN based on clinical evidence from a single randomized trial [9,36].

In a rat model of CIPN, BTZ increased the expression of the transient receptor potential vanilloid type 1 (TRPV1) channel, a nonselective cation channel, and calcitonin gene-related peptide (CGRP), a sensory neuropeptide, in neurons that process nociceptive stimuli, contributing to the development of pain [14]. In a mouse model, BTZ elevated Cav3.2 protein levels and currents in the TTCCs of afferent neurons via the inhibition of proteasome degradation, a pathophysiologic mechanism linked to the development of CIPN [26]. TTCCs are believed to play a role in nociceptive transmission and pain states, as they are highly expressed in the peripheral and central nerve endings of primary sensory neurons and regulate pain signaling [18,20,37,38,39,40,41]. Hence, TTCCs may be a pharmacotherapeutic target for the treatment of neuropathic pain, with potential for the reversal of BTZ-induced CIPN.

In the present studies, suvecaltamide reversed BTZ-induced CIPN in a well-characterized Wistar rat model of chronic BTZ neurotoxicity [30]. The effects of suvecaltamide on BTZ-induced CIPN appear to be dose-dependent across several neurological endpoints (NCV, β-tubulin polymerization, and IENF density). After 4 weeks of co-treatment, suvecaltamide reversed the BTZ-induced reduction of NCV in caudal and sciatic nerves and IENF density in plantar glabrous skin from the hind paws, and attenuated the BTZ-induced reduction elevation of β-tubulin polymerization. The neurotoxicity of BTZ, evaluated by neurophysiological tests, appeared less severe at 5 weeks than at 4 weeks. This apparent discrepancy could be because the statistical analysis performed at 4 weeks included all BTZ-treated animals (i.e., before randomization into four different groups). However, the biological validity of the results is confirmed by the expected increase in BTZ-induced neurotoxicity after 8 weeks of treatment. There was no attenuation of BTZ-induced reduction of MT observed with suvecaltamide. A reduction in acute mechanical sensitivity has been reported in mice and humans treated with TTA-A2, a selective TTCC antagonist [40,42]. It is possible that factors such as treatment duration and/or timing of assessments relative to suvecaltamide administration were not optimal for the evaluation of the effects on MT in this study.

It is important to evaluate whether a pharmacotherapy given in combination with a chemotherapeutic agent interferes with the anti-cancer activity of the chemotherapeutic agent. In the present in vivo and in vitro studies, there was no evidence to suggest that suvecaltamide negatively impacted proteasome inhibition, cytotoxicity, or anti-tumor activity associated with BTZ. In the rat BTZ-induced CIPN model, inhibition of proteasome activity in circulating PBMCs was comparable in rats treated with BTZ alone or co-treated with BTZ+suvecaltamide for 1 or 4 weeks. In cultured MCLs, cytotoxicity (percent cell survival) was similar following exposure to BTZ alone or BTZ+suvecaltamide, and suvecaltamide alone did not induce cytotoxicity. In the xenograft athymic nude mouse tumor model of human myeloma, the tumor volume was similarly reduced with BTZ alone and BTZ+suvecaltamide at day 18. At day 28, the tumor volume was reduced with BTZ+suvecaltamide compared with BTZ alone. In both in vivo studies (rat BTZ-induced CIPN model and xenograft athymic nude mouse tumor model), co-administration of BTZ and suvecaltamide was tolerated with minimal weight loss followed by slight body weight gain, suggesting that combination therapy with BTZ and suvecaltamide does not lead to further weight loss. However, additional studies are warranted to more comprehensively evaluate the safety profile of this drug combination.

Additional experimentation will be required to more fully explore the effects of suvecaltamide on TTCCs as related to BTZ-induced neurotoxicity. Impaired calcium homeostasis and hyperexcitability of TTCCs appear to be key mechanisms underlying BTZ-induced CIPN. BTZ-induced mitochondrial overload of calcium has been reported to cause endoplasmic reticulum stress and result in neurotoxicity [12,43,44,45,46]. TTCCs have been reported to regulate presynaptic glutamate release in dorsal root ganglion neurons [47], and the hyperexcitability of these channels elicited sensitization of pain in BTZ animal models. Calcium overload has also been linked to the BTZ-induced hyperpolymerization of nerve tubulin [33,34], which was reduced by treatment with suvecaltamide in the current study.

The studies reported here have certain limitations, and there are additional areas for further exploration. In the mouse tumor volume study, there was no suvecaltamide alone group to assess the effect of suvecaltamide on tumor growth. In the rat BTZ-induced CIPN study, it would be valuable to determine the effects of a longer treatment duration with suvecaltamide, particularly with regard to measures on which a significant effect of suvecaltamide was not achieved. In addition, the generalizability of these results to peripheral neuropathy induced by other chemotherapeutic agents should be explored. Finally, because these studies were performed only in female animals, it is important to consider whether sex differences in pain perception or neuropathy onset could impact the results in males.

## 5. Conclusions

In conclusion, this preclinical investigation demonstrated that suvecaltamide, a potent and selective modulator of TTCCs, reversed BTZ-induced CIPN without impacting the BTZ anti-cancer activity in rodent models and MCLs. These findings support the further evaluation of TTCCs as a pharmacological target for the treatment of neuropathic pain.

## Data Availability

All relevant data presented in this study are available in this article (Reversal of bortezomib-induced neurotoxicity by suvecaltamide, a selective T-type Ca-channel modulator, in preclinical models) and the Supplementary Material (Table S1: Treatment Group Comparisons and Statistical Analysis of Endpoints in the In Vivo Studies).

## Supplementary Materials

The following is available online at https://www.mdpi.com/article/10.3390/cancers13195013/s1. Table S1: Treatment Group Comparisons and Statistical Analysis of Endpoints in the In Vivo Studies.
