# Supplementary material for: Reversal of Bortezomib-Induced Neurotoxicity by Suvecaltamide, a Selective T-Type Ca-Channel Modulator, in Preclinical Models
Source: Cancers (Basel). 2021 Oct 7;13(19):5013. doi: 10.3390/cancers13195013 (PMC8507761; doi:10.3390/cancers13195013)
Supplement: Supplementary file 1 [file cancers-13-05013-s001.zip › cancers-1359480-supplementary.pdf]

Supplementary Material

Table S1. Treatment Group Comparisons and Statistical Analysis of Endpoints in the *In Vivo* Studies

**NCV OF CAUDAL NERVE (BASELINE) (Figure 1)**	
Mann-Whitney test	
P value	0.4464
Exact or approximate P value?	Gaussian Approximation
One- or two-tailed P value?	Two-tailed
Sum of ranks in column A (CTRL), B (BTZ)	187, 633
Mann-Whitney U	105.0

**NCV OF CAUDAL NERVE (4 WEEKS) (Figure 1)**	
Mann-Whitney test	
P value	0.0029
Exact or approximate P value?	Gaussian Approximation
One- or two-tailed P value?	Two-tailed
Sum of ranks in column A (CTRL), B (BTZ)	74, 136
Mann-Whitney U	0.0000

**NCV OF CAUDAL NERVE (5 WEEKS) (Figure 1)**		
Kruskal-Wallis test		
P value	0.0200	
Dunn's Multiple Comparison Test	Difference in rank sum	P value
CTRL vs BTZ	15.75	P<0.05
CTRL vs BTZ + suvecaltamide 3 mg/kg	4.542	P>0.05
CTRL vs BTZ + suvecaltamide 10 mg/kg	11.13	P>0.05
CTRL vs BTZ + suvecaltamide 30 mg/kg	4.708	P>0.05

BTZ vs BTZ + suvecaltamide 3 mg/kg	-11.21	P>0.05
BTZ vs BTZ + suvecaltamide 10 mg/kg	-4.625	P>0.05
BTZ vs BTZ + suvecaltamide 30 mg/kg	-11.04	P>0.05
BTZ + suvecaltamide 3 mg/kg vs BTZ + suvecaltamide 10 mg/kg	6.583	P>0.05
BTZ + suvecaltamide 3 mg/kg vs BTZ + suvecaltamide 30 mg/kg	0.1667	P>0.05
BTZ + suvecaltamide 10 mg/kg vs BTZ + suvecaltamide 30 mg/kg	-6.417	P>0.05

**NCV OF CAUDAL NERVE (8 WEEKS) (Figure 1)**		
Kruskal-Wallis test		
P value	P<0.0001	
Dunn's Multiple Comparison Test	Difference in rank sum	P value
CTRL vs BTZ	37.00	P<0.001
CTRL vs BTZ + suvecaltamide 3 mg/kg	25.45	P<0.01
CTRL vs BTZ + suvecaltamide 10 mg/kg	14.64	P>0.05
CTRL vs BTZ + suvecaltamide 30 mg/kg	6.583	P>0.05
BTZ vs BTZ + suvecaltamide 3 mg/kg	-11.55	P>0.05
BTZ vs BTZ + suvecaltamide 10 mg/kg	-22.36	P<0.01
BTZ vs BTZ + suvecaltamide 30 mg/kg	-30.42	P<0.001
BTZ + suvecaltamide 3 mg/kg vs BTZ + suvecaltamide 10 mg/kg	-10.82	P>0.05
BTZ + suvecaltamide 3 mg/kg vs BTZ + suvecaltamide 30 mg/kg	-18.87	P<0.05
BTZ + suvecaltamide 10 mg/kg vs BTZ + suvecaltamide 30 mg/kg	-8.053	P>0.05

**NCV OF SCIATIC NERVE (BASELINE) (Figure 2)**	
Mann-Whitney test	
P value	0.1873
Exact or approximate P value?	Gaussian Approximation

One- or two-tailed P value?	Two-tailed
Sum of ranks in column A (CTRL), B (BTZ)	124.5, 695.5
Mann-Whitney U	88.50

**NCV OF SCIATIC NERVE (4 WEEKS) (Figure 2)**	
Mann-Whitney test	
P value	0.0029
Exact or approximate P value?	Gaussian Approximation
One- or two-tailed P value?	Two-tailed
Sum of ranks in column A (CTRL), B (BTZ)	74, 136
Mann-Whitney U	0.0000

**NCV OF SCIATIC NERVE (5 WEEKS) (Figure 2)**		
Kruskal-Wallis test		
P value	0.3513	
Dunn's Multiple Comparison Test	Difference in rank sum	P value
CTRL vs BTZ	2.250	P>0.05
CTRL vs BTZ + suvecaltamide 3 mg/kg	8.917	P>0.05
CTRL vs BTZ + suvecaltamide 10 mg/kg	4.833	P>0.05
CTRL vs BTZ + suvecaltamide 30 mg/kg	7.500	P>0.05
BTZ vs BTZ + suvecaltamide 3 mg/kg	6.667	P>0.05
BTZ vs BTZ + suvecaltamide 10 mg/kg	2.583	P>0.05
BTZ vs BTZ + suvecaltamide 30 mg/kg	5.250	P>0.05
BTZ + suvecaltamide 3 mg/kg vs BTZ + suvecaltamide 10 mg/kg	-4.083	P>0.05
BTZ + suvecaltamide 3 mg/kg vs BTZ + suvecaltamide 30 mg/kg	-1.417	P>0.05
BTZ + suvecaltamide 10 mg/kg vs BTZ + suvecaltamide 30 mg/kg	2.667	P>0.05

NCV OF SCIATIC NERVE (8 WEEKS) (Figure 2)		
Kruskal-Wallis test		
P value	0.0059	
Dunn's Multiple Comparison Test	Difference in rank sum	P value
CTRL vs BTZ	22.10	P<0.05
CTRL vs BTZ + suvecaltamide 3 mg/kg	11.98	P>0.05
CTRL vs BTZ + suvecaltamide 10 mg/kg	2.848	P>0.05
CTRL vs BTZ + suvecaltamide 30 mg/kg	2.333	P>0.05
BTZ vs BTZ + suvecaltamide 3 mg/kg	-10.12	P>0.05
BTZ vs BTZ + suvecaltamide 10 mg/kg	-19.26	P<0.05
BTZ vs BTZ + suvecaltamide 30 mg/kg	-19.77	P<0.05
BTZ + suvecaltamide 3 mg/kg vs BTZ + suvecaltamide 10 mg/kg	-9.136	P>0.05
BTZ + suvecaltamide 3 mg/kg vs BTZ + suvecaltamide 30 mg/kg	-9.652	P>0.05
BTZ + suvecaltamide 10 mg/kg vs BTZ + suvecaltamide 30 mg/kg	-0.5152	P>0.05

MT TEST (BASELINE) (Figure 3)	
Mann-Whitney test	
P value	0.6572
Exact or approximate P value?	Gaussian Approximation
One- or two-tailed P value?	Two-tailed
Sum of ranks in column A (CTRL), B (BTZ)	194, 1184
Mann-Whitney U	158.0

MT TEST (4 WEEKS) (Figure 3)	
Mann-Whitney test	
P value	0.0001
Exact or approximate P value?	Gaussian Approximation

One- or two-tailed P value?	Two-tailed
Sum of ranks in column A (CTRL), B (BTZ)	322.5, 758.5
Mann-Whitney U	17.50

**MT TEST (5 WEEKS) (Figure 3)**		
Kruskal-Wallis test		
P value	0.0065	
Dunn's Multiple Comparison Test	Difference in rank sum	P value
CTRL vs BTZ	17.24	P<0.05
CTRL vs BTZ + suvecaltamide 3 mg/kg	3.250	P>0.05
CTRL vs BTZ + suvecaltamide 10 mg/kg	16.50	P<0.05
CTRL vs BTZ + suvecaltamide 30 mg/kg	10.56	P>0.05
BTZ vs BTZ + suvecaltamide 3 mg/kg	-13.99	P>0.05
BTZ vs BTZ + suvecaltamide 10 mg/kg	-0.7411	P>0.05
BTZ vs BTZ + suvecaltamide 30 mg/kg	-6.679	P>0.05
BTZ + suvecaltamide 3 mg/kg vs BTZ + suvecaltamide 10 mg/kg	13.25	P>0.05
BTZ + suvecaltamide 3 mg/kg vs BTZ + suvecaltamide 30 mg/kg	7.313	P>0.05
BTZ + suvecaltamide 10 mg/kg vs BTZ + suvecaltamide 30 mg/kg	-5.938	P>0.05

**MT TEST (8 WEEKS) (Figure 3)**		
Kruskal-Wallis test		
P value	0.0011	
Dunn's Multiple Comparison Test	Difference in rank sum	P value
CTRL vs BTZ	23.19	P<0.001
CTRL vs BTZ + suvecaltamide 3 mg/kg	7.563	P>0.05
CTRL vs BTZ + suvecaltamide 10 mg/kg	16.69	P<0.05

CTRL vs BTZ + suvecaltamide 30 mg/kg	13.50	P>0.05
BTZ vs BTZ + suvecaltamide 3 mg/kg	-15.63	P>0.05
BTZ vs BTZ + suvecaltamide 10 mg/kg	-6.500	P>0.05
BTZ vs BTZ + suvecaltamide 30 mg/kg	-9.688	P>0.05
BTZ + suvecaltamide 3 mg/kg vs BTZ + suvecaltamide 10 mg/kg	9.125	P>0.05
BTZ + suvecaltamide 3 mg/kg vs BTZ + suvecaltamide 30 mg/kg	5.938	P>0.05
BTZ + suvecaltamide 10 mg/kg vs BTZ + suvecaltamide 30 mg/kg	-3.188	P>0.05

**β-TUBULIN POLYMERIZATION (Figure 4A)**		
Kruskal-Wallis test		
P value	0.0003	
Dunn's Multiple Comparison Test	Difference in rank sum	P value
CTRL vs BTZ	-11.88	P<0.05
CTRL vs BTZ + suvecaltamide 3 mg/kg	-15.63	P<0.05
CTRL vs BTZ + suvecaltamide 10 mg/kg	-16.13	P<0.05
CTRL vs BTZ + suvecaltamide 30 mg/kg	0.3750	P>0.05
BTZ vs BTZ + suvecaltamide 3 mg/kg	-3.750	P>0.05
BTZ vs BTZ + suvecaltamide 10 mg/kg	-4.250	P>0.05
BTZ vs BTZ + suvecaltamide 30 mg/kg	12.25	P>0.05
BTZ + suvecaltamide 3 mg/kg vs BTZ + suvecaltamide 10 mg/kg	-0.5000	P>0.05
BTZ + suvecaltamide 3 mg/kg vs BTZ + suvecaltamide 30 mg/kg	16.00	P>0.05
BTZ + suvecaltamide 10 mg/kg vs BTZ + suvecaltamide 30 mg/kg	16.50	P<0.05

**IENF DENSITY (Figure 4B)**		
Kruskal-Wallis test		
P value	P<0.0001	

Dunn's Multiple Comparison Test	Difference in rank sum	P value
CTRL vs BTZ	28.99	P<0.001
CTRL vs BTZ + suvecaltamide 3 mg/kg	18.06	P<0.05
CTRL vs BTZ + suvecaltamide 10 mg/kg	28.94	P<0.001
CTRL vs BTZ + suvecaltamide 30 mg/kg	8.444	P>0.05
BTZ vs BTZ + suvecaltamide 3 mg/kg	-10.93	P>0.05
BTZ vs BTZ + suvecaltamide 10 mg/kg	-0.04167	P>0.05
BTZ vs BTZ + suvecaltamide 30 mg/kg	-20.54	P<0.05
BTZ + suvecaltamide 3 mg/kg vs BTZ + suvecaltamide 10 mg/kg	10.89	P>0.05
BTZ + suvecaltamide 3 mg/kg vs BTZ + suvecaltamide 30 mg/kg	-9.611	P>0.05
BTZ + suvecaltamide 10 mg/kg vs BTZ + suvecaltamide 30 mg/kg	-20.50	P<0.01

**PROTEASOME INHIBITION (BASELINE) vs BTZ (4 WEEKS) (Figure 5A)**	
Mann-Whitney test	
P value	0.0286
Exact or approximate P value?	Exact
One- or two-tailed P value?	Two-tailed
Sum of ranks in column A (BTZ baseline), B (BTZ 4 weeks)	10, 26
Mann-Whitney U	0.0000

**TUMOR GROWTH (DAY 18) (Figure 5C right)**		
Kruskal-Wallis test		
P value	0.0005	
Dunn's Multiple Comparison Test	Difference in rank sum	P value
Vehicle Control vs BTZ alone	13.13	P<0.001
Vehicle Control vs BTZ + suvecaltamide	10.13	P<0.05
BTZ alone vs BTZ + suvecaltamide	-3.000	P>0.05

TUMOR GROWTH (DAY 28) (Figure 5C right)	
Mann-Whitney test	
P value	0.0070
Exact or approximate P value?	Gaussian Approximation
One- or two-tailed P value?	Two-tailed
Sum of ranks in column A (BTZ), B ()	92.50, 43.50
Mann-Whitney U	7.500

BTZ = bortezomib; CTRL = control; IENF = intraepidermal nerve fiber; NCV = nerve conduction velocity.

Due to no adjustments for multiplicity, the P values presented are nominal.
